# Blocking pathogen transmission at the source: reservoir targeted OspA-based vaccines against *Borrelia burgdorferi*

**DOI:** 10.3389/fcimb.2014.00136

**Published:** 2014-09-26

**Authors:** Maria Gomes-Solecki

**Affiliations:** Department of Microbiology, Immunology and Biochemistry, University of Tennessee Health Science CenterMemphis, TN, USA

**Keywords:** *Borrelia burgdorferi*, Lyme disease, enzootic cycle, oral vaccination, transmission cycles, wildlife reservoir

## Abstract

Control strategies are especially challenging for microbial diseases caused by pathogens that persist in wildlife reservoirs and use arthropod vectors to cycle amongst those species. One of the most relevant illnesses that pose a direct human health risk is Lyme disease; in the US, the Centers for Disease Control and Prevention recently revised the probable number of cases by 10-fold, to 300,000 cases per year. Caused by *Borrelia burgdorferi*, Lyme disease can affect the nervous system, joints and heart. No human vaccine is approved by the Food and Drug Administration. In addition to novel human vaccines, new strategies for prevention of Lyme disease consist of pest management interventions, vector-targeted vaccines and reservoir-targeted vaccines. However, even human vaccines can not prevent Lyme disease expansion into other geographical areas. The other strategies aim at reducing tick density and at disrupting the transmission of *B. burgdorferi* by targeting one or more key elements that maintain the enzootic cycle: the reservoir host and/or the tick vector. Here, I provide a brief overview of the application of an OspA-based wildlife reservoir targeted vaccine aimed at reducing transmission of *B. burgdorferi* and present it as a strategy for reducing Lyme disease risk to humans.

Given the unabated increase in Lyme disease in the US, public health officials are searching for effective and creative solutions to quell the epidemic. The immunization of animals as a means of preventing disease in humans and in domesticated animals of agricultural economic interest was comprehensively reviewed by Monath (2013). The use of reservoir targeted vaccines to control disease is an attractive but under-explored concept. This approach benefits primarily zoonotic diseases in which wild animals play a major role in transmitting the pathogen to humans such as plague, rabies and Lyme disease but it can be easily applied to other vector-borne and directly transmitted diseases. Here my goal is to provide an update on progress toward a wildlife targeted vaccine as a means to reduce risk of exposure to Lyme disease, while discussing its strengths, limitations and a framework for its application.

## *Borrelia burgdorferi* and lyme disease

Lyme disease was clinically characterized after an epidemic of asymmetrical arthritis affecting multiple joints was observed in children in Lyme, Connecticut, US in 1977 (Steere et al., 1977). The disease has steadily increased in incidence (Kugeler et al., 2008) and has expanded its geographic range (Kugeler et al., 2008; Diuk-Wasser et al., 2012). Caused by a spirochete, *B. burgdorferi* sensu stricto (s.s.) (Benach et al., 1983; Steere et al., 1983), Lyme disease accounts for >90% of all vector-borne diseases with nearly 30,000 confirmed cases reported each year to the Center for Disease Control and Prevention (CDC). Recently the CDC revised the number of probable infections upward 10-fold (to 300,000) to account for wide underreporting (http://www.cdc.gov/lyme/) (Young, 1998).

Lyme disease spirochetes are transmitted to reservoir hosts by hard-bodied ticks (Burgdorfer, 1989). Because the *B. burgdorferi* genome does not encode any known toxins or the machinery that would be required to secrete them (Fraser et al., 1997), tissue damage and disease are mediated by the inflammatory response that follows infection in the mammalian host (Weis and Bockenstedt, 2010). Erythema migrans is the most common clinical manifestation of *B. burgdorferi* infection (60–80%) (Stanek et al., 2012) and develops after an incubation period of 2–32 days (Wormser et al., 2006). Most cases of erythema migrans occur between June and August (Kugeler et al., 2008). Low level spirochetemia occurs in ~50% of untreated patients presenting with signs of early Lyme disease (Wormser et al., 2005). Occasionally, Lyme disease affects the peripheral or central nervous system, joints or heart months after infection (Steere, 1989).

## The enzootic cycle and transmission

Transmission of *B. burgdorferi* occurs in rural areas used for forestry and recreational activities and in peridomestic suburban areas (Piesman and Gern, 2004; Stanek et al., 2012). Development and survival of the ticks depends on the level of humidity provided by a layer of decaying vegetation in the understory of deciduous or mixed woodlands which provide support and shelter to a range of vertebrate reservoir hosts that sustain transmission of the bacterium (Stanek et al., 2012) in states endemic for Lyme disease.

*Borrelia burgdorferi* is transmitted by at least four species of tick within the *Ixodes ricinus-persulcatus* complex: *I. scapularis* and *I. pacificus* in eastern and western North America, respectively; *I. ricinus* in Europe, and *I. persulcatus* in Asia (Burgdorfer, 1989). These ticks go through a four-stage life cycle (egg, larvae, nymph, and adult) feeding only once per active stage. Unfed ticks attach to the skin of a host as the animal passes through vegetation. After feeding for 3–7 days the ticks drop off their host and take shelter under leaf litter where they need a minimum relative humidity of 80% to survive (Piesman and Gern, 2004). They take several months to molt into the next stage of development (Stanek et al., 2012). Larvae are uninfected as they hatch—there is no transovarial transmission (Patrican, 1997)—and *B. burgdorferi* is acquired upon feeding on an infected reservoir host. After molting to the nymphal stage, the tick transmits the pathogen to a susceptible animal or human providing its next blood meal. Transmission of Lyme borreliae occurs through injection of tick saliva during feeding (Ribeiro et al., 1987). A feeding period of at least 36 h is needed for transmission of *B. burgdorferi* by *I. scapularis* or *I. pacificus* ticks (Peavey and Lane, 1995; des Vignes et al., 2001) and infection becomes increasingly likely after the tick has been attached for 48 h (Piesman et al., 1987).

The life cycle of all four species of ticks have distinct seasonality. *I. scapularis* nymphs are active in early summer and adults become active in the fall, remain so until winter and become active again in the spring; furthermore, after the fall and winter, nymphal ticks from the following tick cohort undergo questing in the spring (Piesman and Gern, 2004). In the case of *I. ricinus* and *I. persulcatus*, nymphs and adults become active in early spring and continue to seek hosts until mid-summer; both stages can be active until later in the year in sheltered humid environments. With *I. ricinus* a second peak of activity can occur in the fall. Patterns of activity of *I. pacificus*, prevalent in the western US, seem more like those of *I. ricinus* than *I. scapularis* (Piesman and Gern, 2004). In all species, peak activity usually occurs slightly later in larvae than in nymphs, especially for *I. scapularis* in the eastern US. The 3 month difference between one cohort of *I. scapularis* nymphal activity and the following cohort of larval activity allows plenty of time for transmission from infected reservoir hosts to larvae and could explain the high transmission rate in the eastern US (Eisen et al., 2002). Most transmission to humans occurs from May to September, coinciding with the activity of nymphs and with the increasing recreational use of tick habitats by the public (Kugeler et al., 2008; Stanek et al., 2012).

Field studies in North America and Eurasia have identified a variety of small-mammal and avian reservoirs in enzootic transmission cycles (Table 1). The white-footed mouse, *Peromyscus leucopus*, is considered to be the main reservoir in the northeastern US (Anderson, 1989). In most tick habitats, deer are essential for the maintenance of the tick population (Anderson, 1989) because they are one of the few wild hosts that can feed sufficient numbers of adult ticks and keep the cycle ongoing, but they are not competent reservoirs for spirochetes (Matuschka et al., 1993). Cattle are non-competent hosts and sheep also appear to be non-competent reservoirs (Matuschka et al., 1993; Ogden et al., 1997). Different pathogenic genospecies of *B burgdorferi* sensu lato favor some vertebrates as reservoir hosts, though this host specificity is not absolute. One factor thought to be relevant to reservoir competence is the resistance of the particular genospecies of Lyme borreliae to complement-mediated killing by the animal host (Bykowski et al., 2008). Populations of deer in a tick habitat can be regarded as a good indication of Lyme disease risk because an array of other hosts, including reservoir competent animals, are likely to be present. If most animals in a habitat are those which do not act as reservoirs for Lyme borreliae, such as deer or cattle, Lyme disease risk may decrease because ticks will feed mostly on these animals and therefore will reinforce a positive feedback loop by not becoming infected (LoGiudice et al., 2003). However, this is a highly debated issue. The presence of diversionary hosts may reduce the proportion of ticks infected, but may actually increase the overall abundance of infected ticks because they augment the entire tick population, allowing more larvae to feed on reservoir competent hosts vs. a smaller population (Ogden and Tsao, 2009; Mannelli et al., 2012; Randolph and Dobson, 2012; Wood and Lafferty, 2013). The specific effect probably varies with the hosts involved and with the degree non-competent hosts “out-compete” the reservoir hosts for juvenile ticks (Ogden and Tsao, 2009).

**Table 1:** **The reservoir competence of vertebrate species**.

	**Species**	**Geographical area**	**References**
Competent	Mouse	US/Europe	De Boer et al., 1993; LoGiudice et al., 2003
	Voles	Europe	De Boer et al., 1993
	Chipmunk	US	LoGiudice et al., 2003
	Shrew	US	LoGiudice et al., 2003; Dykhuizen et al., 2008
	Squirrel	US/Europe	LoGiudice et al., 2003; Mannelli et al., 2012
	Ground birds	US/Europe	LoGiudice et al., 2003; Mannelli et al., 2012
	Striped skunk	US	LoGiudice et al., 2003
Weakly/Non-competent	Deer	US/Europe	Gray et al., 1992; LoGiudice et al., 2003
	Cattle	Europe	Mannelli et al., 2012
	Opossum	US	LoGiudice et al., 2003
	Raccoon	US	LoGiudice et al., 2003
	Lizards	US/Europe	Casher et al., 2002; Eisen et al., 2004; Salkeld and Lane, 2010; De Sousa et al., 2012
	Catbirds	US	Mather et al., 1989; Ginsberg et al., 2005
	Sheep	Europe	Gray et al., 1995; Ogden et al., 1997
Not confirmed	Hare	Europe	Jaenson and Talleklint, 1996
	Hedgehog	Europe	Gern et al., 1997
	Badgers	Europe	Mannelli et al., 2012
	Red foxes	Europe	Mannelli et al., 2012

*Reservoir competence is the ability of a host to become infected, remain systemically infected and then transmit the pathogen to a feeding vector (Gray et al., 2002); from a population level, it is the probability that an infected host transmits B. burgdorferi to feeding ticks (Brunner et al., 2008). Reservoir competence is the product of (1) the probability the host is infected (infection prevalence), and (2) the probability an infected host will transmit the infection to a feeding vector (infectivity). Prevalence varies in space and time, whereas infectivity is a property of the host species and it depends on the strain of B. burgdorferi*.

## The human OspA vaccine and other methods of prevention

There are multiple strategies for prevention of Lyme disease. Direct vaccination of humans could have been the gold standard for prevention of Lyme disease, as vaccination in general is the gold standard for prevention of a multitude of infectious diseases. A vaccine based on OspA protected mice against *B. burgdorferi* infection after tick challenge (Fikrig et al., 1992a,b; de Silva et al., 1996); this work led to successful clinical trials that culminated in the approval of the human vaccine by the FDA in 1998. Individuals vaccinated subcutaneously showed approximately ~76% protection against *B. burgdorferi* infection after receiving three vaccine doses (Steere et al., 1998). Despite adequate efficacy results, the human vaccine was removed from the market. Other vaccines based on assorted combinations of four to eight OspC types appear to be promising candidates as preventive measures against Lyme disease (Seinost et al., 1999; Earnhart and Marconi, 2007; Earnhart et al., 2007).

However, disease expansion into new geographical areas (Burgdorfer, 1989; Ogden et al., 2005; Kugeler et al., 2008; Mannelli et al., 2012) can not be controlled by promoting only direct human vaccination programs. Given that humans are only incidental hosts of *B. burgdorferi*, even mandatory human vaccination will not reduce *B. burgdorferi* from its enzootic cycle; *B. burgdorferi* will simply cycle through its reservoir hosts and vectors until optimal environmental conditions and breaches in vaccine effectiveness allow for its recurrence. Integrated approaches that build upon effective methods to contain *B. burgdorferi* infection within its enzootic cycle as well as direct vaccination of humans would lead to a synergistic effect in public health more likely to yield sustainable positive outcomes (Monath, 2013).

## Control of tick density

Human risk of Lyme disease from an environmental perspective is measured in terms of density of infected nymphs (Diuk-Wasser et al., 2012). Other methods of tick control have been explored to reduce the risk of Lyme disease ranging from avoiding tick infested environs, covering exposed skin, using tick repellents, bathing within 2 h of tick exposure, to daily inspections of the entire skin surface to remove attached ticks (Connally et al., 2009), to chemoprophylaxis after removal of attached ticks (Wormser et al., 2007; Warshafsky et al., 2010). Management of wood chips where lawns are adjacent to forests, application of acaricides, and the construction of fences to keep out deer are also effective, as these measures disturb the habitat where the density of host seeking ticks is high (Piesman, 2006). Acaricide self-treatment of white-tailed deer resulted in reduction of tick density (Hoen et al., 2009; Stafford et al., 2009) and a rodent-targeted acaricide (fipronil) delivered to white-footed mice (*P. leucopus*) in modified commercial bait boxes was also effective in reducing nymphal and larval tick infestations as it reduced tick density (Dolan et al., 2004). A decrease in tick density is expected to reduce human exposure to Lyme disease as it would reduce density of infected ticks.

## Reservoir targeted vaccines

Vaccines targeted to the host reservoir have been developed as strategies to reduce transmission of pathogens (Piesman, 2006). Baits and baiting systems have proven effective for delivery of rabies and plague vaccines (Pastoret et al., 1988; Creekmore et al., 2002; Knobel et al., 2002). An example of a successful application is the oral baited vaccine Raboral™ currently used by local governments in the United States to create barriers between infected wildlife and highly populated areas to prevent transmission of rabies (Blanton et al., 2012).

*Ixodes scapularis and I. pacificus* ticks and its competent reservoirs are distributed across the US in many areas where Lyme disease is not endemic as appears to be the case in California (Eisen et al., 2009) and in the Southern states of the US (Oliver et al., 2003). In the presence of both competent reservoir and tick vector, one reason for the lack of Lyme disease in these areas could be that environmental weather conditions dramatically affects the ecology of the habitat that sustains the enzootic cycle of *B. burgdorferi* and enable pathogen transmission (Eisen et al., 2002). Another way to achieve the same goal would be to remove *B. burgdorferi* from the enzootic cycle through the application of reservoir targeted vaccines.

Although eradicating the main reservoir host(s), deer and the vector are measures generally accepted as effective in disrupting transmission of *B. burgdorferi*, the fact that the competent reservoir and vector co-exist in areas where *B. burdgorferi* does not appear to be transmitted to humans argues against the necessity of implementing drastic measures to eliminate any. It further argues in favor of a solution that is less disruptive of an ecosystem that sustain life and lifestyles. Thus, preventing the vector from acquiring or transmitting an organism may be an effective strategy for preventing Lyme disease in humans (Clark and Hu, 2008). Although elimination of Lyme borreliae from nature is unrealistic, diminishing their threat to humans is an achievable goal (Radolf et al., 2012).

**Figure 1:**
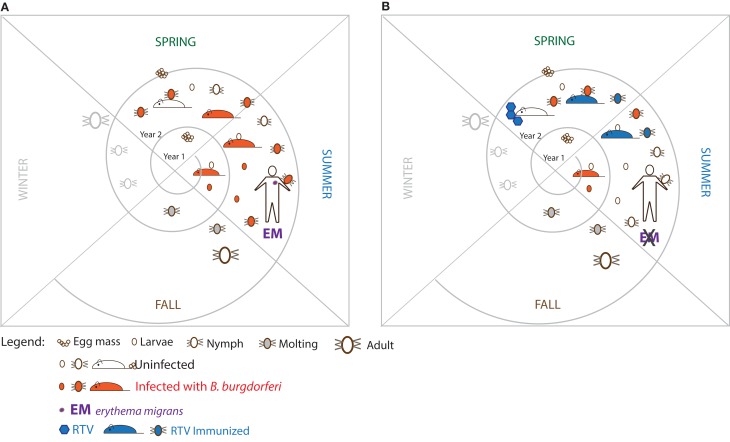
**Proposed strategy to break the enzootic cycle of the Lyme disease spirochete. (A)** The triad which comprises the enzootic cycle: the tick vector (*Ixodes* sp.), the major reservoir host (white-footed mouse) and *Borrelia burgdorferi*. **(B)** Hypothesis: immunizing wild white-footed mice with oral reservoir targeted vaccine (RTV) could break the enzootic cycle of *B. burgdorferi*.

The reservoir host that is most competent for transmission of *B. burgdorferi* in the US is the white-footed mouse (*Peromyscus leucopus*) (Anderson et al., 1987; Anderson, 1989). However, chipmunks, squirrels, shrews, and other small vertebrates are increasingly recognized as important hosts (Brisson et al., 2008). Further, birds may also play a role in spreading *B. burgdorferi* (Burgdorfer, 1989; Comstedt et al., 2006; Ogden et al., 2010). The principle behind development of reservoir targeted vaccines (RTV) is to reduce *B. burgdorferi* from the reservoir host(s) and from the ticks that feed on them. In other words, the ultimate objective of RTV vaccination is to turn competent reservoir hosts into incompetent hosts while at the same time depleting *B. burgdorferi* from the vector without eradicating either (the vertebrate host or the tick) from the ecosystem (Figure 1).

OspA was previously used to vaccinate mice orally (Fikrig et al., 1991; Luke et al., 1997). Two groups have been developing baited OspA-based oral delivery systems to vaccinate reservoir hosts against Lyme disease. One delivery system is based on Vaccinia virus (VV) (Scheckelhoff et al., 2006) and the other is based on *E. coli* expressing OspA (Gomes-Solecki et al., 2006). Both systems are equally effective in eliciting production of protective levels of anti-OspA antibodies in inbred *M. musculus* and in outbred *P. leucopus* mice that receive the vaccine orally either via oral gavage or via *ad libitum* feeding. Furthermore, both vaccines are equally effective in decreasing *B. burgdorferi* from infected ticks that fed on vaccinated mice (Gomes-Solecki et al., 2006; Scheckelhoff et al., 2006; Bhattacharya et al., 2011; Meirelles Richer et al., 2011). While VV-based vaccines require a desirable low number of doses to induce protective immune responses in the host, they are also potentially infectious to people who are immunocompromised or suffer from eczema (Reed et al., 2012). The bacteria-based delivery system, on the other hand, requires that a higher number of vaccine doses be deployed to induce protective immune responses in the host, which burdens any distribution plan, but it is also considered a safer approach. In an alternative approach, a doxycycline rodent bait formulation prevented tick transmission of *B. burgdorferi* to vertebrate hosts as well as cured established infections in mice (Dolan et al., 2008; Zeidner et al., 2008). A decrease in tick infection with *B. burgdorferi*, as well as the decrease of vertebrate host infection is expected to result in an overall decrease in human risk of Lyme disease.

A 1-year field study testing the effect of a reservoir targeted vaccine in Lyme disease risk was done in Connecticut in 1998 and it was repeated in 2002 (Tsao et al., 2004). Mice were trapped, injected subcutaneously with an OspA-based suspension, and in the following year ticks were collected in immediate surrounding areas to test for infection with *B. burgdorferi*. The year after deployment of the RTV, nymphal infection prevalence was reduced by ~24%. A comprehensive 5-year field study of an oral RTV against *B. burgdorferi* was reported recently (Richer et al., 2014). The second study differed from the first in vaccine formulation (oral bait delivery of vaccine comprised of bacteria previously induced to produce OspA) and duration of vaccine treatment (treatment up to 5 years). The field study results demonstrated that a bacteria-based oral bait vaccine delivered yearly to trapped wild white-footed mice resulted in reduced infection rates of nymphal ticks the year after RTV deployment (23%) and that 5 years of consecutive treatment caused a substantial disruption of the enzootic cycle of *B. burgdorferi* with reductions in nymphal infection prevalence as high as 76%. The VV based RTV was not tested in field studies. These data offer empirical support to an analysis using a dynamic model of *B. burgdorferi* transmission which predicted that mouse-reservoir vaccination was expected to reduce infection prevalence amongst ticks by 56% (Tsao et al., 2012).

Such decreases in the prevalence of infected *I. scapularis* vectors could significantly reduce the risk to humans and other accidental mammalian hosts (such as dogs) of acquiring Lyme disease. Implementation of a reservoir targeted vaccine (RTV) as part of integrated pest management interventions to block transmission of *B. burgdorferi* should consider deployment both in sylvatic as well as in suburban areas and broad distribution of such vaccines should take into account issues of toxicity to humans.

## Strengths, limitations, and framework for application

A major strength of a reservoir targeted vaccine approach is to bypass immunizing humans instantly avoiding potential vaccine failures and side effects (Monath, 2013); additionally, for Lyme disease, the RTV objective is to remove only the pathogen (*B. burgdorferi*) from the ecosystem while leaving all other living components of the enzootic cycle undisturbed (Richer et al., 2014). RTVs control infections acquired from wild animals, which leads to collaborations between animal and human health industries that, in turn, should lead to implementation of more robust public health measures. Furthermore, accelerated regulatory pathways should lead to faster licensing of new vaccines (Monath, 2013).

One of the limitations of the anti-*Borrelia* RTVs described is that both are based in a single immunogen, OspA, which neutralizes the spirochete in the tick midgut (Fikrig et al., 1992a,b; de Silva et al., 1996). A vaccine based on OspA supplemented with assorted OspC types (Earnhart and Marconi, 2007; Earnhart et al., 2007) may be more efficacious given that targeting both the vector (OspA) and the host (OspC) would contribute to protection. Only one of the reservoir species that contribute *B. burgdorferi* to the enzootic cycle—mice—was targeted for treatment (Tsao et al., 2004; Gomes-Solecki et al., 2006; Scheckelhoff et al., 2006; Dolan et al., 2008; Zeidner et al., 2008; Bhattacharya et al., 2011; Meirelles Richer et al., 2011; Richer et al., 2014). Other small vertebrates such as chipmunks, squirrels, and shrews also transmit *B. burgdorferi* effectively to the tick (LoGiudice et al., 2003; Dykhuizen et al., 2008; Mannelli et al., 2012) and should be included in integrated pest management interventions. RTVs are not designed to reduce tick density, which is considered to be an important eco-epidemiological measure of Lyme disease risk (Ogden and Tsao, 2009; Mannelli et al., 2012). A multi-pronged integrated pest management intervention comprised of RTV treatment to reduce pathogen burden in the ticks supplemented by acaricide treatment to reduce tick density and by vaccination of humans at risk of infection maybe the most effective route to drastically reduce incidence of Lyme disease in human populations.

## Summary

Reservoir targeted vaccines are under development for prevention of transmission of *B. burgdorferi* as a means to reduce Lyme disease risk. Oral vaccines administered to mice using virus-based or bacteria-based delivery vehicles expressing OspA reduced *B. burdorferi* from the tick vector in the laboratory. Dynamic models of *B. burgdorferi* transmission predicted that mouse-reservoir vaccination was expected to reduce infection prevalence amongst ticks by 56%. OspA based RTVs tested in short-term (1-year) and long-term (5-year) field trials lead to reductions of tick infection prevalence ranging from 24 to 76%, respectively. Other reservoir targeted approaches reduced infection prevalence in the tick vector as well as cured infection in the reservoir host. A multi-pronged integrated pest management intervention combined with human vaccination maybe the most effective route to drastically reduce Lyme disease incidence. Lastly, eco-epidemiological factors should be studied to understand the relationship between the pathogen, the tick, the hosts, and its environs to be able to assess the long-term effects of any strategies that may affect the ecosystem.

### Conflict of interest statement

Maria Gomes-Solecki has a relevant patent and is a co-founder of US BIOLOGIC.
